# The Behavior of Beams Reinforced with Patches Under Three-Point Bending: An Experimental Investigation

**DOI:** 10.3390/polym17222993

**Published:** 2025-11-11

**Authors:** Fatima Benaoum, Foudil Khelil, Abdelghani Baltach, Demet Ulku Gulpinar Sekban, Ecren Uzun Yaylacı, Ali Benhamena, Mohamed Mouli, Dursun Murat Sekban, Murat Yaylacı

**Affiliations:** 1 Mechanical Engineering Department, University of Mascara, Mascara 29000, Algeria; fatima.benaoum@univ-mascara.dz (F.B.); khelilfoudil@yahoo.com (F.K.); ali_benhamena@yahoo.fr (A.B.); 2 Mechanical Engineering Department, University of Tiaret, Tiaret 14035, Algeria; abdelghani.baltach@univ-tiaret.dz; 3 Department of Landscape Architecture, Karadeniz Technical University, Trabzon 61080, Türkiye; demetsekban@ktu.edu.tr; 4 Faculty of Fisheries, Recep Tayyip Erdogan University, Rize 53100, Türkiye; ecren.uzunyaylaci@erdogan.edu.tr; 5 LABMAT Materials Laboratory, ENPO Maurice Audin, Oran 31000, Algeria; moulimohamed@yahoo.fr; 6 Department of Marine Engineering Operations, Karadeniz Technical University, Trabzon 61080, Türkiye; msekban@ktu.edu.tr; 7 Department of Civil Engineering, Recep Tayyip Erdogan University, Rize 53100, Türkiye; 8 Faculty of Turgut Kıran Maritime, Recep Tayyip Erdogan University, Rize 53900, Türkiye

**Keywords:** carbon fiber, crack, strengthening, flexural behavior, ductility

## Abstract

This study assesses the flexural performance of concrete beams repaired with externally bonded carbon-fiber-reinforced polymer (CFRP) patches under controlled damage conditions. Prismatic beams (7 × 7 × 28 cm) underwent three-point bending tests in four configurations: uncracked, uncracked-reinforced, cracked-unrepaired, and cracked-repaired. Pre-existing damage was caused by mid-span notches at a = 7, 21, and 35 mm. CFRP patches were placed on the tension face, and the ultimate load and failure mode were recorded. Repairing CFRP beams increased maximum load by up to 240% compared to unrepaired counterparts, and the failure characteristic changed from brittle shear to ductile flexural. Strengthening uncracked beams also yielded significant benefits. These findings show that patch-type CFRP reinforcement effectively recovers and enhances flexural performance across a wide range of crack severity, and they provide quantitative guidelines for determining repair levels depending on original crack depth.

## 1. Introduction

The rehabilitation of existing structures using composite materials has emerged as a highly effective solution in modern construction. Among these materials, carbon fiber-reinforced polymer (CFRP) composites stand out due to their exceptional mechanical properties, lightweight nature, and resistance to corrosion and environmental degradation. These features make CFRP ideal for strengthening and extending the lifespan of structures in civil engineering applications, including bridges, buildings, marine infrastructure, landscape components, and urban infrastructure [1,2,3].

CFRP consists of woven carbon fibers embedded in a polymer matrix, offering a high strength-to-weight ratio and durability under harsh conditions [4,5,6,7]. Their adaptability to various forms and weaving patterns also enables aesthetically pleasing and structurally efficient designs [8,9]. Beyond conventional architectural uses, these features make CFRP suitable for diverse applications, including facade retrofitting, public space installations, and landscape architectural elements, where visual harmony and mechanical performance are both essential. Due to these advantages, CFRP has been widely adopted for retrofitting and reinforcing concrete and steel structures, improving load-bearing capacity and structural integrity [10,11,12,13,14].

Numerous studies have highlighted CFRP’s performance in structural applications. The contribution of CFRP to the structural performance of concrete elements has been well documented. Ozturk et al. [15] reported that CFRP significantly improves the load-carrying capacity and ductility of retrofitted geopolymer concrete beams. Similarly, Zhou et al. [16] confirmed the effectiveness of CFRP in enhancing the seismic performance of circular RC bridge piers under lateral impact loading. Meizhong et al. [17] demonstrated that replacing steel stirrups with CFRP strips improved fatigue life in concrete beams. Similarly, Dong et al. [18] found that CFRP-concrete-filled steel tube (CFRP-CFST) piles exhibited enhanced mechanical and corrosion resistance in humid, high-temperature environments.

Other research has focused on corrosion resistance and structural performance under various stress conditions. Ananthkumar et al. [19] demonstrated the effectiveness of CFRP and GFRP in mitigating rebar corrosion. Despite their advantages, CFRP composites also present limitations, including high costs, brittleness under certain conditions, and limited fire resistance [20,21]. Still, their increasing use in seismic strengthening, bridge retrofitting, and concrete reinforcement reflects their potential for future civil engineering innovations [22,23,24].

In this way, CFRP helps extend the lifespan and enhance the safety of buildings, particularly by strengthening concrete beams. It can be used as external reinforcement or lamination to improve flexural strength, and ductility, and reduce reliance on traditional reinforcement, while also preventing premature failure from corrosion. Experiments with drop hammer impact and three-point bending tests on CFRP-strengthened RC beams showed that hammer height and CFRP bonding influenced the dynamic response and failure patterns, with strain cracks observed using high-speed cameras and digital image correlation [25]. Zhu et al. [26] studied the behavior of CFRP-reinforced concrete beams using electromechanical impedance (EMI)-based monitoring to assess interfacial performance under sustained loading and wet-dry cycles. Qiang et al. [27] tested composite beams with CFRP plates to analyze their bending behavior, showing that CFRP reinforcement significantly improves stiffness and load-bearing capacity. Yu et al. [28] proposed failure modes based on data from CFRP-reinforced concrete beams, while Wang et al. [29] used a numerical model to analyze the behavior of CFRP-reinforced steel beams under load. Guo et al. [30] examined the effect of temperature on the deformation and debonding behavior of CFRP-reinforced beams. Finally, Liu et al. [31] conducted a finite element analysis to evaluate the long-term behavior of CFRP-reinforced recycled concrete beams, considering various factors such as tendon relaxation and concrete creep.

Also, Jin et al. [32] developed a 3D mesoscale simulation method to analyze failure mechanisms in CFRP-reinforced concrete beams, finding that increasing stirrup and CFRP fiber ratios improves strength but reduces nominal shear strength as section size increases. Huang et al. [33] created a device to predict the static and flexural behavior of H-type reinforced concrete beams strengthened with CFRP bars, measuring ultimate resistance and ductility improvements through a 3D nonlinear finite element model. Lastly, Lam et al. [34] investigated methods to reinforce materials against web buckling in steel beams using CFRP plates.

To address all the factors surrounding the issue, the researchers conducted experimental analyses on four single-coped steel beams, both with and without CFRP reinforcement. They found that CFRP plates significantly enhance load-bearing capacity, especially when multiple layers are used, and this was analyzed using a finite element model (FEM). Zhang et al. [35] demonstrated the strengthening effects of CFRP in both conventional reinforced concrete and CFRP-strengthened beams through quasi-static loading and drop hammer impact tests. Their findings indicated that CFRP sheets reduce damage in the mid-span of beams, minimize residual displacement, and help in the removal of impact damage. Additional studies show CFRP’s effectiveness in preventing web buckling in steel beams, reducing impact damage, and enhancing durability in prefabricated components [35,36,37,38,39,40]. These findings underline CFRP’s crucial role in improving both new and existing civil infrastructure. Gemi et al. [36,37] used CFRP composites to strengthen prefabricated purlins against shear damage from vertical loading, noting that bending damage was the primary failure mode and that vertical loading capacity increased by up to 59% depending on CFRP wrapping. Ozkilic et al. [40] investigated the behavior of prefabricated concrete purlins (PCPs) using finite element modeling in ABAQUS, examining various parameters such as reinforcement ratios and CFRP properties. They concluded that the effects of CFRP-related parameters were more significant than those related to reinforced concrete. Finally, Ozkilic et al. [39] examined reinforced concrete beams with circular holes and CFRP-strengthened failures, shear-deficient, under-balanced reinforced concrete beams with rectangular cross-sections.

This paper aims to explore the mechanical behavior of CFRP-reinforced concrete beams using a repair method based on bonded composite plates. Given the rising costs associated with structural rehabilitation, this topic has garnered significant attention. The study includes quality control of concrete, testing procedures, and interpretation of compressive strength data. Twenty-four test beams were examined using a three-point bending setup to assess stress–strain relationships, failure modes, and load-bearing capacity. The research specifically focuses on the flexural behavior of these beams, analyzing parameters such as stress and strain, load and deformation, failure modes, load capacity, and crack size. The findings are intended to serve as a benchmark for evaluating similar structural elements under varying damage conditions, including those found in architectural, infrastructural, and landscape design contexts.

Although many studies have been conducted in the literature on composite materials [41,42,43,44,45], most existing research focuses on externally bonded reinforcement applied under uniform or undamaged conditions. In contrast, the present study investigates the behavior of CFRP-repaired concrete beams that were intentionally pre-damaged with predefined crack lengths. This experimental approach allows for a systematic evaluation of CFRP repair efficiency as a function of damage severity, an area still underexplored in the literature. Therefore, this work offers a new perspective on the application of CFRP for localized rehabilitation of cracked concrete beams.

## 2. Experimental Procedure

A multiphase experimental investigation was carried out by the authors. The study involved two groups of beams: the first tested in a prevailing compressive load regime and the second tested in a prevailing three-point bending load regime. The concrete beams were mixed, cast, and cured in the laboratory using the Faury method [46], with the concrete supplied by a mixing station. Fresh concrete was evaluated for slump, slump flow, air content, and density, as summarized in Table 1. Measurements for fresh concrete properties (Table 1) were carried out with standard laboratory equipment. The reported values correspond to the mean of repeated readings, with a resolution of ±0.01 t/m^3^ for density and ±1 min for setting time.

**Figure 1:**
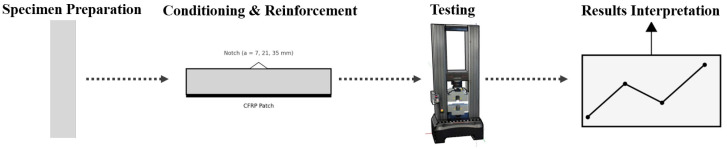
Schematic flowchart of specimen preparation, reinforcement, testing, and analysis steps.

Initially, measurements were taken of mixed ingredients (cement, sand, and water) with properties listed in the first table. The quantities are prepared and then mixed with the concrete mixer for 90 s. Samples were demolded after 24 h and stored in water until testing, which was conducted at 28 days. While these values are generally stable, even slight variations in temperature and humidity could influence the performance of concrete and CFRP, especially over extended testing periods. It is important to ensure that these environmental factors are closely monitored and maintained to guarantee consistent results. The working steps are shown in Figure 1.

The concrete beams were tested under controlled laboratory conditions of approximately 20 °C room temperature and 45% relative humidity, which are considered typical values for ensuring consistency and minimizing the influence of environmental factors on the performance of both concrete and CFRP.

### 2.1. The Preparation of Cylindrical Specimens with Dimensions

The preparation of cylindrical specimens measuring 16 cm × 32 cm (160 mm in diameter × 320 mm in height) is a crucial step to experimentally determine the compressive strength of concrete. The preparation of these specimens is carried out in several stages. First, the inside of the molds is cleaned and oiled to facilitate demolding. Fresh concrete is then poured in three equal layers. Each layer is carefully compacted, either manually using a metal rod or mechanically with a vibrating needle or vibrating table, to avoid air bubbles. Once the mold is filled, the surface is leveled with a trowel. The samples are left to rest for 24 h at room temperature, covered with plastic film or a wet cloth. After this period, they are removed from the mold and placed in a water bath at 20 ± 2 °C for 28 days, until the test day. It should be noted that curing is the process used to prevent premature water loss from concrete. It helps prevent rapid hardening of the concrete during the drying phase and acts as a waterproof, impermeable layer on the surface.

After 24 h, the concrete begins to harden on the surface, but it is still far from reaching its ultimate strength due to its brittleness. However, after 28 days of storage in water, it reaches 100% of its capacity and is fully usable. Concrete is usually tested between seven and 28 days after curing. After seven days, concrete typically shows two-thirds of its expected strength, and because it almost reaches its expected ultimate strength after 28 days, this is a good early indicator of its ultimate strength. This justifies our choice of a 28-day storage period in water. After this period, it is dried and ready for testing.

### 2.2. Interpretation of Compressive Strength Test Results

The purpose of the test was to determine the compressive strength at 28 days (fc28), which must be ≥25 MPa to validate the concrete’s conformity to class C25/30. The compression crushing test involved applying increasing force until cracks appeared, with the pressure at failure corresponding to the concrete’s strength.

**Figure 2:**
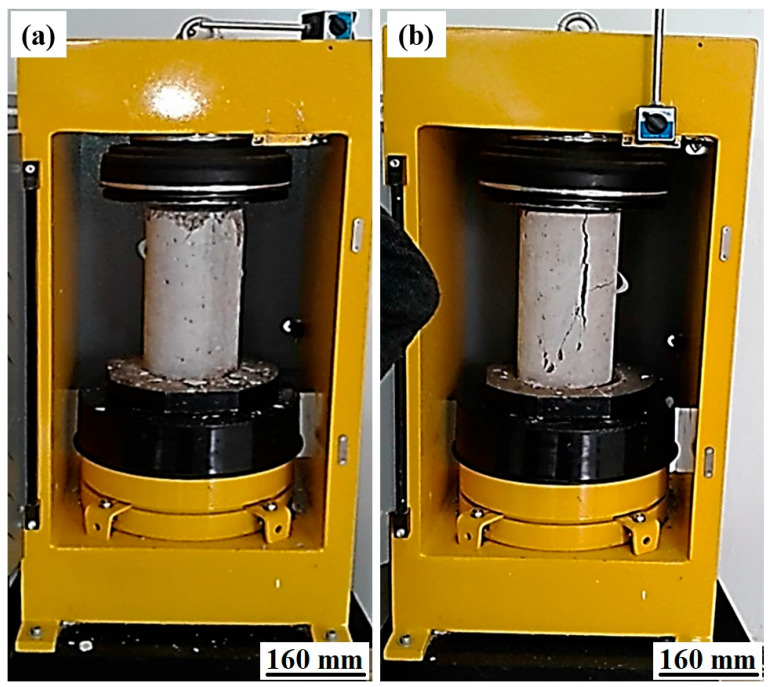
RP 3000 XP compression testing machine: (**a**) concrete specimens during compressive testing and (**b**) concrete specimens after the compression test.

The rupture of the test pieces is most often of the “rupture by longitudinal column” type (see Figure 2b), sometimes by bursting. The appearance of the fracture surface is rough to smooth, revealing some weakness of the matrix. This research demonstrates the feasibility of producing BHP High-Performance Concrete using locally available materials. Particularly noteworthy is the utilization of CPA 325 cement, distinguished by its high alumina content.

## 3. Results and Discussions

### 3.1. Compressive Strength Evolution

**Figure 3:**
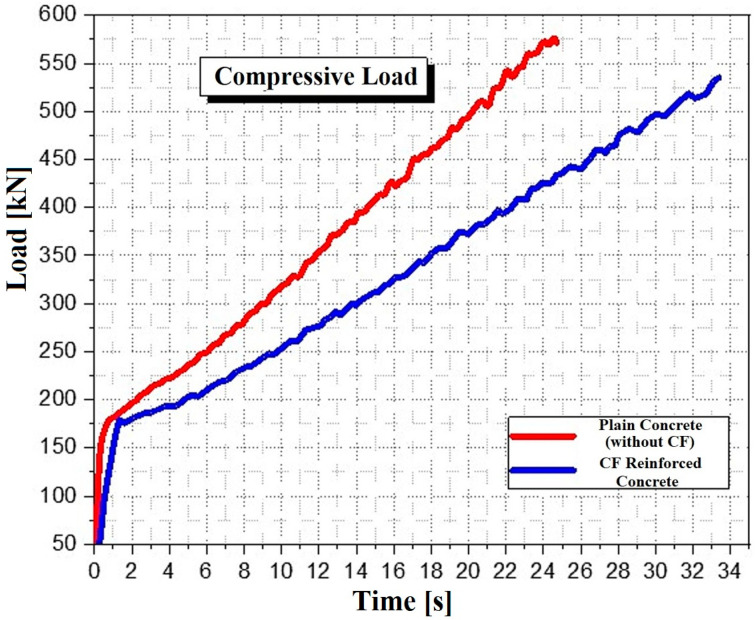
Compressive loading behavior of plain concrete and CF reinforced concrete.

Figure 3 shows the change in compressive resistance over time during the compression tests. The strength of cylindrical concrete specimens without carbon fiber (CF) increased with time, with resistances ranging between 533.5 kN and 582.21 kN (26.7 MPa and 29.10 MPa). The compressive resistance stabilized at around 25 to 34 s, likely due to improved slip resistance of preexisting microcracks, reducing crack growth energy. Proper preparation of concrete with small gravel aggregates also enhanced fracture toughness by bridging cracks. These findings are consistent with previous studies [47,48]. Additionally, mid-span failures of beams in the first series were characterized by concrete crushing in the mid-level compression zone, validating their design to withstand compressive failure.

### 3.2. Formability Behavior

This study falls within this context and aims to evaluate the effectiveness of applying carbon fiber patches to concrete beams through a three-point bending test. The objective is to assess the improvement provided by these reinforcements in terms of bending strength, post-cracking behavior, and overall load-bearing capacity.

To achieve this, concrete specimens of standard dimensions (7 cm × 7 cm × 28 cm) were manufactured. They were designed and prepared using the same composition and method as those described in the experimental procedure section, similar to the cylindrical specimens, some of which were reinforced with carbon fiber patches applied by manual lamination. These samples were then subjected to a three-point bending test, allowing for a comparative analysis of the reinforcement’s impact on the structural performance of the beams.

#### 3.2.1. Crack Geometry and Notched Specimen Preparation

In this study, certain specimens were intentionally notched prior to testing in order to simulate pre-existing cracks and assess the effectiveness of carbon fiber-reinforced polymer (CFRP) patch reinforcement under controlled damage conditions. These notches were carefully and consistently introduced during the casting process by inserting a 1 mm thick plexiglass plate at the center of the mold, perpendicular to the longest axis of the prismatic specimens (7 cm × 7 cm × 28 cm). This method produced well-defined crack depths of 7 mm, 21 mm, and 35 mm, denoted in the manuscript by the variable a, with values expressed in millimeters. This setup ensured uniform crack geometry across all samples, which is essential for accurate and reproducible evaluation of the mechanical performance of both reinforced and unreinforced beams.

#### 3.2.2. Beams Reinforced with Carbon Fiber

In our method, carbon fiber patches are bonded to the concrete beam through manual lamination. The procedure begins with preparing the concrete surface, followed by the application of a layer of epoxy adhesive using a roller to ensure an even coating. Subsequently, unidirectional carbon fibers are positioned, and a roller is used to straighten the fibers while eliminating larger air pockets. Before bonding, the cracked surfaces were cleaned and slightly roughened, the epoxy resin was applied uniformly, and the CFRP strips were pressed firmly to ensure proper anchorage. The properties of the carbon fiber patch material are detailed in Table 2. Carbon fiber properties (Table 2) are taken directly from the manufacturer’s datasheet; therefore, statistical spread is not applicable, and values are presented as nominal datasheet values.

In this section, the focus is on the impact of the carbon fiber patch on the reinforcement of beams subjected to three-point bending. The aim is to investigate the role of the carbon fiber patch in enhancing the beam’s resistance to bending. Bending experiments were conducted on beams that were cast with characterized concrete.

In order to highlight the role and effectiveness of external reinforcement by carbon fiber patch in a predominantly bending loading regime, seven beams (B2 series and B3 series) were designed to be identical in all aspects of composition, with the exception of their geometric shape, test loading regime, and the external reinforcement. The beams did not have any internal reinforcement.

#### 3.2.3. Patching Specimens Before Testing

**Figure 4:**
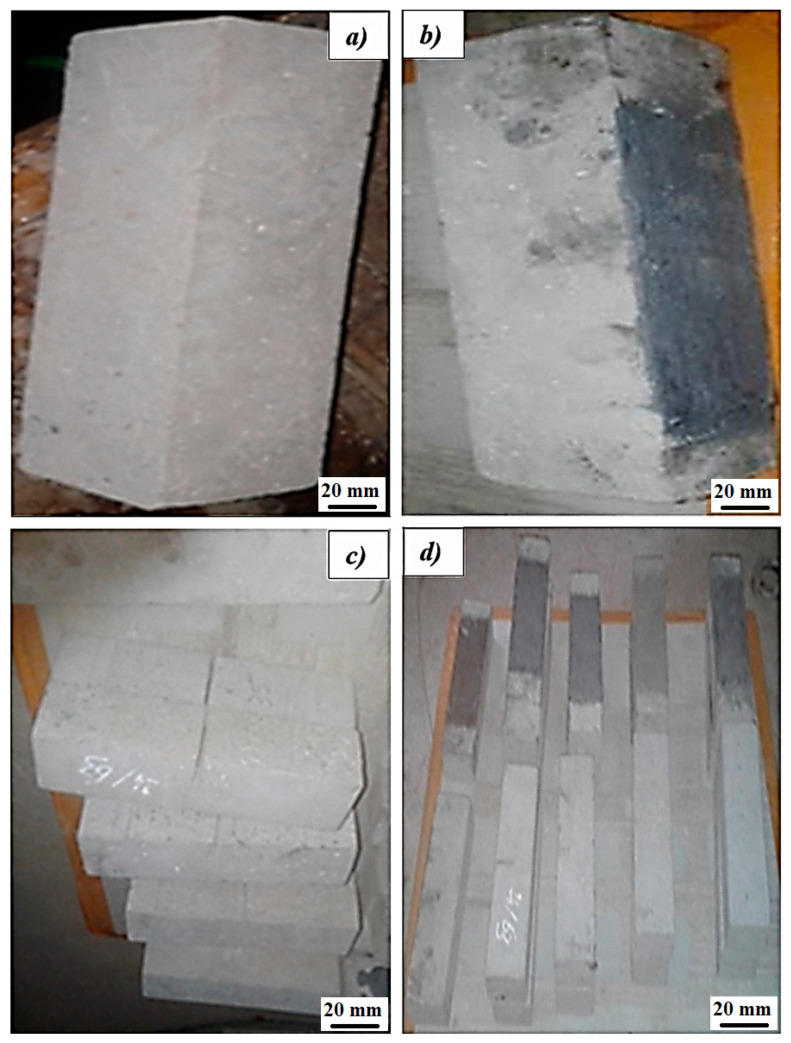
Structural classification of samples under different conditions: (**a**) uncracked samples, (**b**) uncracked sample reinforced with carbon fiber patches, (**c**) cracked sample cracked during forming, and (**d**) cracked sample repaired with carbon fiber patches.

In this study, specimens were prepared to compare their mechanical behavior under flexion. Figure 4 shows details of the different specimens prepared for testing.

The CFRP reinforcement patches have a rectangular section of 180 mm × 70 mm and a thickness of approximately 1.5 mm. This reinforcement covers 64% of the span of the specimens. These patches were prepared and bonded to the bottom part of the specimen after surface cleaning. The surface was roughened to expose the aggregate layer, a thin layer of epoxy was spread evenly, and the fibers were pressed onto the beam using a roller to remove air voids.

In each group of beams, three layers of longitudinal carbon fiber were used. The laminate area was demarcated, and needle scrapers were used to roughen and remove the top layer of concrete, exposing the first layer of aggregate. The thickness of the bonding resin is an important parameter for the best performance of the reinforcement system. A very small thickness can compromise the bonding of the laminate, while a high thickness can increase the stress concentrations in the area of the laminate anchoring. Therefore, according to the recommendations of the manufacturers, a resin thickness of 1–2 mm was applied.

To fulfill the initial conditions mentioned above for each type of beam, the chronological sequence of the experimental program is fixed, as shown in Table 3. The above-mentioned sequential tests evaluated the effect of repairing cracks in beams before bonding CFRP laminates.

It should be noted that three specimens were tested per group. Although this number limits the ability to compute standard deviations or confidence intervals, all specimens were prepared using the same mix proportions, casting procedures, and curing conditions. The resulting values showed minimal variation across specimens, indicating consistent performance.

#### 3.2.4. Flexural Test Configuration

**Figure 5:**
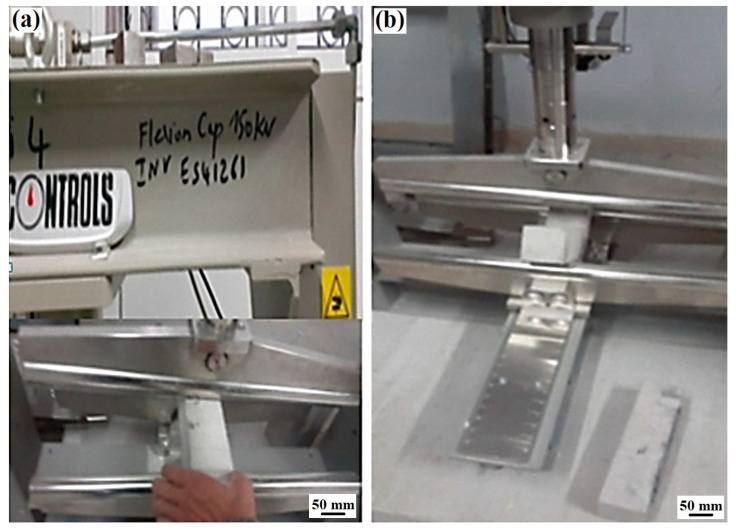
Bending testing machine on beam specimens: (**a**) specimen mounted in the bending beam creep tester and (**b**) view of specimen after flexural test.

In this study, the specimens were prepared to compare their mechanical behavior under static load. Figure 5 presents the details of the tested specimens.

A testing apparatus was employed, utilizing a Controls machine with a 150 kN capacity, featuring dual test modes: longitudinal and transverse, which are particularly suited for FRC experiments. The maximum distance between the lower rollers is 1 m. Bending loading at three points was ensured using a jack, with the maximum moment occurring at the midpoint. The supports are positioned 35 mm from the beam ends.

The load application was centralized at the midpoint of the beam spans under study (Figure 5). Tests were conducted with underload control, maintaining an average loading rate of 0.05 kN/s.

All reinforced concrete beams underwent fabrication and testing under three-point bending until failure in a monotonic manner.

**Figure 6:**
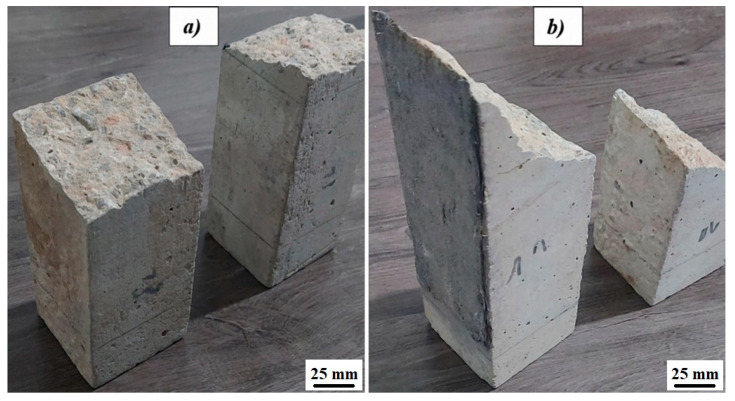
Failure profiles of specimens: (**a**) B1 and (**b**) B4.

To begin, we start with the first case, where reference control specimens, series B, were subjected to a bending load until failure. The objective was to evaluate the bending behavior (stiffness and strength) of the unreinforced beams and to determine the damage load they experienced. Figure 6 compares the failure of two specimens: one composed of uncracked, unreinforced beams (B1) and the other reinforced (B4).

Comparing these two models shows that crack damage occurs much faster in the unreinforced specimens than in the reinforced ones. Upon comparing these two profiles, it is notable that crack damage occurs much more rapidly in the unreinforced specimens compared to the reinforced ones.

**Figure 7:**
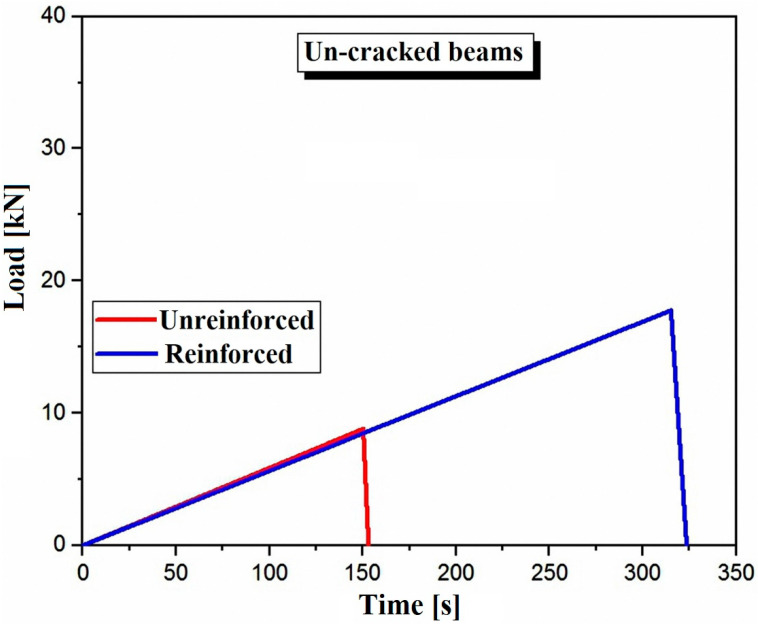
Comparison of the ultimate load-bearing capacity of the tested beam specimens B1 and B4 under three-point bending.

This observation is confirmed by the failure path, where the path of the unreinforced specimens is perpendicular to the neutral axis of the beam, which is associated with a very short time to failure. Conversely, the failure path of the reinforced specimens is oblique in relation to the neutral axis of the beam, resulting in a higher time to failure and stress. Figure 7 compares the time to failure of a reinforced beam of family B’ and another unreinforced beam of family B. This figure indicates that crack damage occurs more quickly in case A, where the crack direction is vertical, representing the shortest distance and reflecting the beam thickness. In contrast, in case B, the crack path is inclined at a 45-degree angle to the vertical, which increases the distance and thus doubles the time to failure, delaying it more compared to the first case.

We observe a significant increase in the time to failure for the reinforced beam compared to the unreinforced beam. This increase can go as far as doubling the time to failure. For the reinforced beam, the ultimate load reaches 17.46 kN, while for the unreinforced beam, it is only 8.24 kN, approximately half of the value recorded for the reinforced beam.

**Figure 8:**
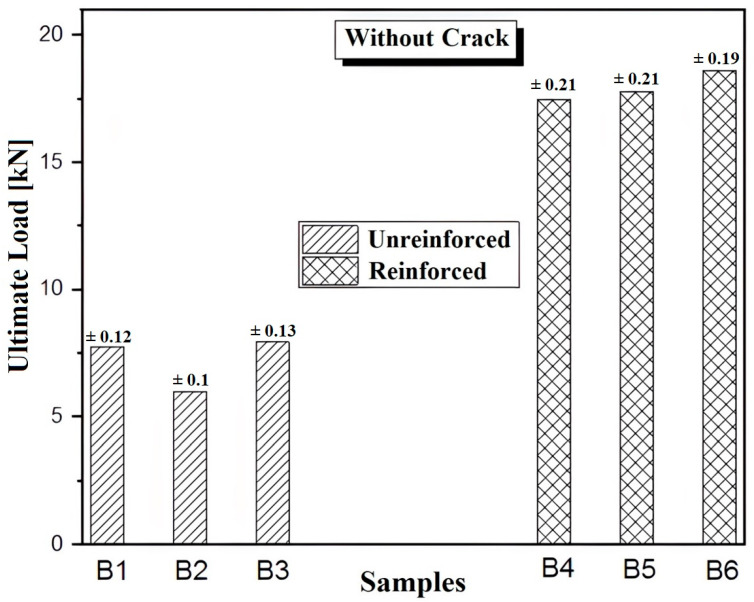
Comparison of ultimate load for unreinforced and reinforced samples.

The previous figure (Figure 8) compares the cumulative ultimate load for series B1, B2, and B3, representing unreinforced and uncracked samples, series B4, B5, and B6, representing unreinforced uncracked specimens, and series B4, B5, and B6, representing reinforced uncracked specimens. According to this figure, it can be observed that for each specimen in series B, the ultimate load varies between 6.53 and 8.62 kN, whereas for specimens in series B, the ultimate load varies between 19 and 20.25 kN. In addition, reinforcement with CFRP patches significantly increases the ultimate load and consequently prolongs the lifespan of the reinforced structure. As a result, the ultimate load recorded for the reinforced specimens is higher than that of the unreinforced specimens, reaching up to 200%.

Furthermore, in case 2, an assessment was conducted to analyze the influence of CFRP reinforcement on beams from series C, D, and E. These series feature cracks of varying lengths, 7 mm, 21 mm, and 35 mm, positioned at the midpoint of the span.

Identical mixtures and conditions were employed for these series as were used for the preceding ones. It should be noted that each serial model has three samples.

**Figure 9:**
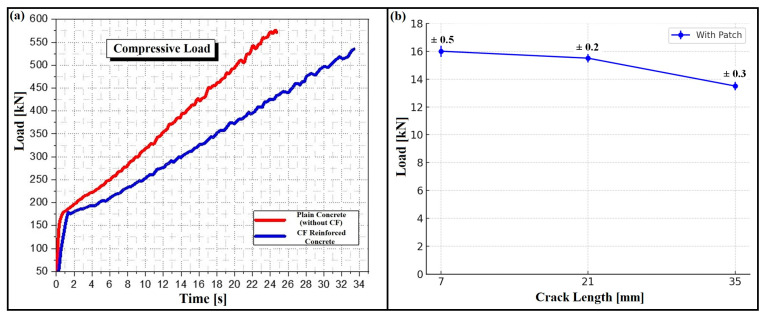
(**a**) Load–time curves of plain and CF-reinforced concrete, (**b**) ultimate load versus crack length for repaired beams with error bars.

In this section, unrepaired cracked specimens will be examined. The experimental results are illustrated in Figure 9a, which shows the variation in ultimate loads for the series of unrepaired cracked specimens subjected to three-point bending.

It can be observed that the current results are nearly similar for each series with the same crack length and that an increase in this length leads to a significant decrease in ultimate load and consequently a reduction in the lifespan of the cracked structure.

These examples now support the statement and reinforce the validity of the claim. For this purpose, Figure 9b represents beams in series C’, D’, and E’, which were repaired with CFRP.

Figure 9 shows that patch reinforcement significantly increases the ultimate load threshold. As the crack length increases, an asymptotic behavior of crack paths is observed for all patched configurations. This trend is noted for all three samples in each series.

Although the test results clearly indicate that CFRP reinforcement significantly enhances the flexural strength of concrete beams, the manuscript would benefit from a more in-depth exploration of the mechanisms responsible for these improvements. CFRP significantly enhances the structural performance of concrete by compensating for its inherent weakness in tension. When applied to the tension zone of a beam, CFRP bears a substantial portion of the tensile stresses, thereby redistributing internal forces and delaying both the initiation and propagation of cracks.

Beyond increasing tensile capacity, CFRP also improves crack behavior by bridging existing cracks and limiting their growth. This leads to better crack control and promotes a more ductile failure mode, ultimately improving the beam’s serviceability and durability. Additionally, the tensile stiffness of the CFRP layer can shift the neutral axis upward, enhancing flexural stiffness and resulting in increased load-bearing capacity.

**Figure 10:**
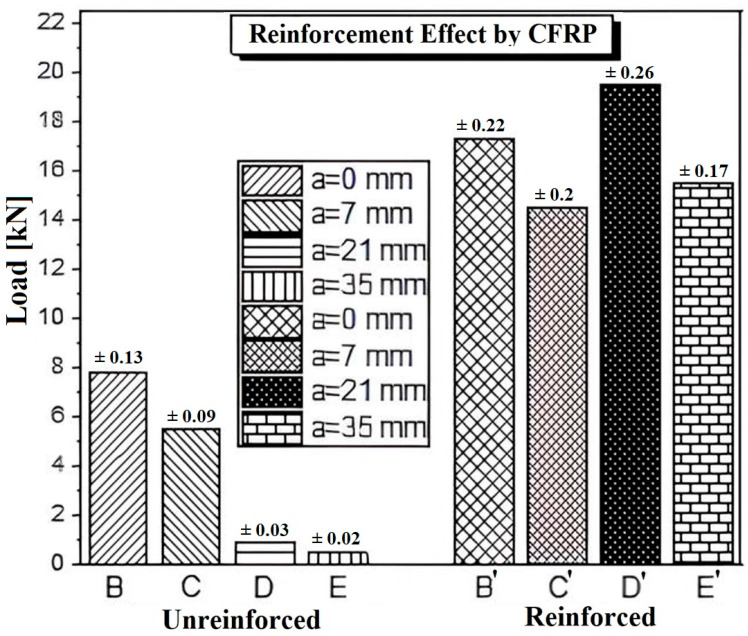
Comparison of load capacity for unreinforced and CFRP reinforced beams with different crack depths.

For better clarification, we present the values of the ultimate load thresholds of all the studied series (case 1 and case 2) in Figure 10. In Figure 10, for each series of specimens, we calculated the average ultimate load threshold of the three samples. Then, all the analyzed series are compared. The analysis of Figure 10 shows that the force stress thresholds decrease with increasing crack length for all the unrepaired series, while the repaired series exhibit similar trends, especially for the cracked series.

The results obtained clearly show that beams reinforced with glued carbon fibers exhibit significantly higher ultimate load thresholds compared to unrepaired concrete. This effect, particularly pronounced in Series E (up to 20 times higher), highlights the importance of effective bonding between CFRP and concrete, as adequate adhesion ensures efficient stress transfer. The addition of carbon fibers, therefore, plays a crucial role in enhancing the ductility of high-strength concrete, even with minimal bonding.

This finding is especially relevant as it highlights the effectiveness of carbon fibers in reinforcing concrete, particularly by increasing ductility and resistance to damage. Beyond this, reinforcement extends the lifespan of concrete structures and improves their capacity to withstand high loads. The dramatic increase observed in Series E’, up to 20 times higher, demonstrates the considerable potential of carbon fibers to enhance the mechanical performance of high-strength concrete.

**Figure 11:**
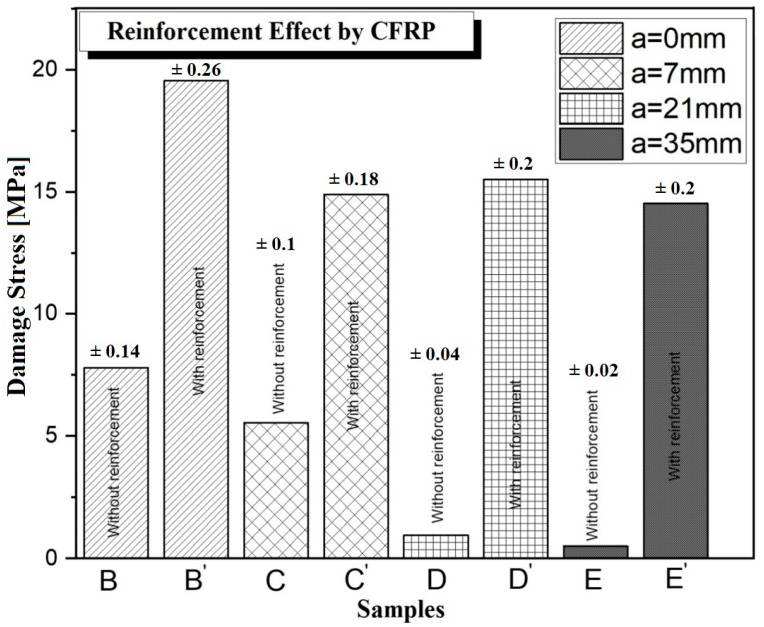
Comparison of damage stress for unreinforced and CFRP reinforced beams with different crack depths.

As shown in Figure 11, the length of cracks in specimens repaired with carbon fibers appears to influence the material’s ability to distribute loads and limit crack propagation. Shorter cracks enable better adhesion and force transmission, whereas longer cracks reduce the effectiveness of the repair if load transfer is not adequately ensured. Based on the results, CFRP reinforcement remains effective for cracks of 7 mm and 21 mm, but its contribution becomes less significant at around 35 mm, where the reinforcement effect is limited.

It should also be noted that this study focused on cracks located at the mid-span, corresponding to the maximum bending moment region. If cracks were located closer to the supports, the impact on bearing capacity would likely differ, as shear effects become more dominant and could reduce the efficiency of CFRP reinforcement.

Finally, this study highlights the importance of considering several key factors when designing an effective CFRP repair system. These include the initial length of cracks before repair, the distribution and density of fibers, the repair method used (such as gluing or injection), and the orientation of the fibers to ensure proper anchorage. Addressing these elements can significantly improve the performance and durability of rehabilitated structural elements.

## 4. Conclusions

The use of composite patches made of carbon fibers offers significant advantages in terms of high strength, homogeneity, and micro-filling, particularly in the repair of micro-cracks in concrete. Experimental results demonstrate superior performance in terms of strength and durability for reinforced specimens (B’, C’, D’, and E’) compared to those that were not reinforced (B, C, D, and E). The results obtained from the study are summarized below.

1. Reinforcement with CFRP significantly improves the efficiency of uncracked lightweight concrete beams, with gains ranging from 130% to 190% compared to non-reinforced beams.
2. For cracked beams, the load-carrying capacity increased significantly, with gains of 170% to 240% for cracks of 7 mm in length, and even higher values for cracks of 21 mm and 35 mm after reinforcement.
3. The reinforced beams typically failed in bending without any detachment of the CFRP sheets, indicating effective bonding, whereas the non-reinforced beams generally failed through shear cracking and concrete crushing, reflecting lower structural integrity”.
4. Thus, reinforcement using composite patches proves to be an effective method for enhancing the mechanical performance and durability of concrete structures, particularly in applications requiring localized repairs or an increase in load-carrying capacity.

## Figures and Tables

**Table 1:** Properties and dosage for 1 m^3^ of fresh concrete used.

Cement		Coarse Aggregate		Mass Composition of Concrete		
Property	Value	Property	Value	Element	Percent (%)	Mass (kg)
Fineness	18%	Class	Gravel 3/8	Gravel 3/8	47.40%	1100
Initial setting time	30 min	Absolute density	2.665 t/m^3^	Sand	26.72%	620
Soundness	0.233	Apparent volumetric mass	1.38 t/m^3^	Cement	17.24%	400
Standard consistency	34%	Coefficient of form	0.138	Water	8.62%	200.02

**Table 2:** Mechanical properties of carbon fiber [49].

Properties	Value
Density, ρ (kg/m^3^)	1700
Elastic modulus, E1 (GPa)	235
Elastic modulus, E2 (GPa)	155
Poison’s ratio, ν12	0.25
Longitudinal tensile strength, XT (MPa)	3590
Longitudinal compressive strength, XC (MPa)	950
Transverse tensile strength, YT (MPa)	117
Transverse compressive strength, YC (MPa)	367

**Table 3:** Experimental series.

Series	Specimens	Crack	Reinforcement
Reference B	B1, B2 and B3	No	No
Strengthened B’	B4, B5 and B6	No	Yes
Cracked (crack length a = 7 mm) C	C1, C2 and C3	Yes	No
Repaired and strengthened (crack length a = 7 mm) C’	C4, C5 and C6	Yes	Yes
Cracked (crack length a = 21 mm) D	D1, D2 and D3	Yes	No
Repaired and strengthened crack length (a = 21 mm) D’	D4, D5 and D6	Yes	Yes
Cracked (crack length a = 35 mm) E	E1, E2 and E3	Yes	No
Repaired and strengthened (a = 35 mm) E’	E4, E5 and E6	Yes	Yes

## Data Availability

The original contributions presented in this study are included in the article. Further inquiries can be directed to the corresponding author.
